# Supplementary material for: In Vivo Imaging of Acute Hindlimb Ischaemia in Rat Model: A Pre-Clinical PET Study
Source: Pharmaceutics. 2024 Apr 15;16(4):542. doi: 10.3390/pharmaceutics16040542 (PMC11054801; doi:10.3390/pharmaceutics16040542)
Supplement: Supplementary file 1 [file pharmaceutics-16-00542-s001.zip › pharmaceutics-2908206-supplementary.pdf]

Supplementary Figure S1.

The complete (unedited) Western blot images provided in the manuscript.

APN/CD 13

1 2 3 4 5 6 7 8 9

150 kDa

150 kDa

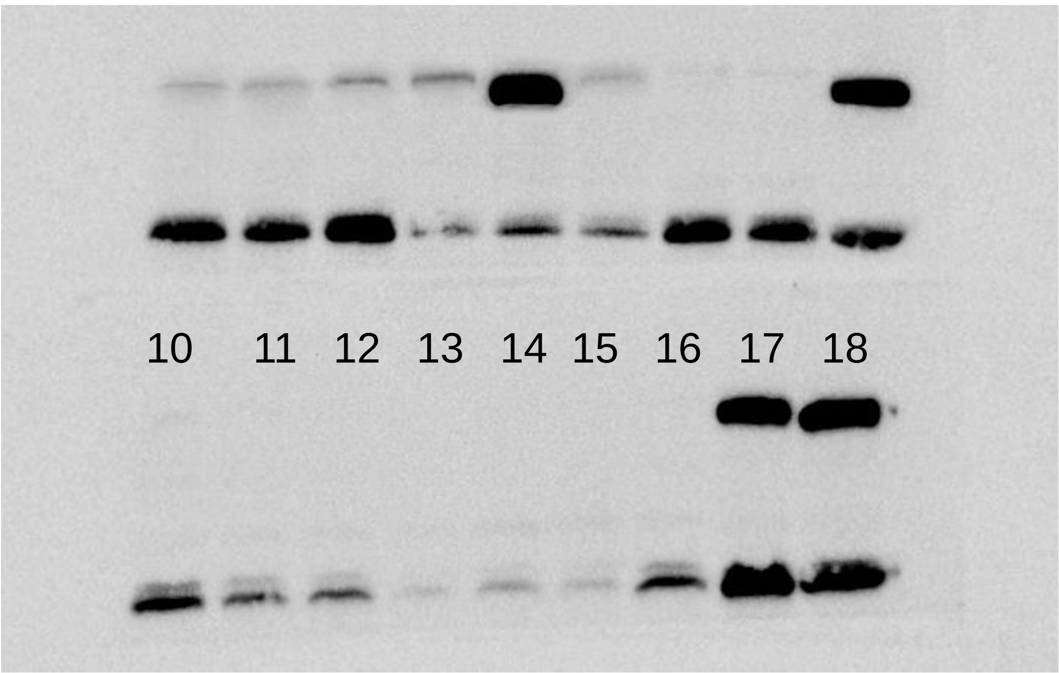

CD13

CD13

HPRT housekeeping gene

1 2 3 4 5 6 7 8 9

25 kDa

25 kDa

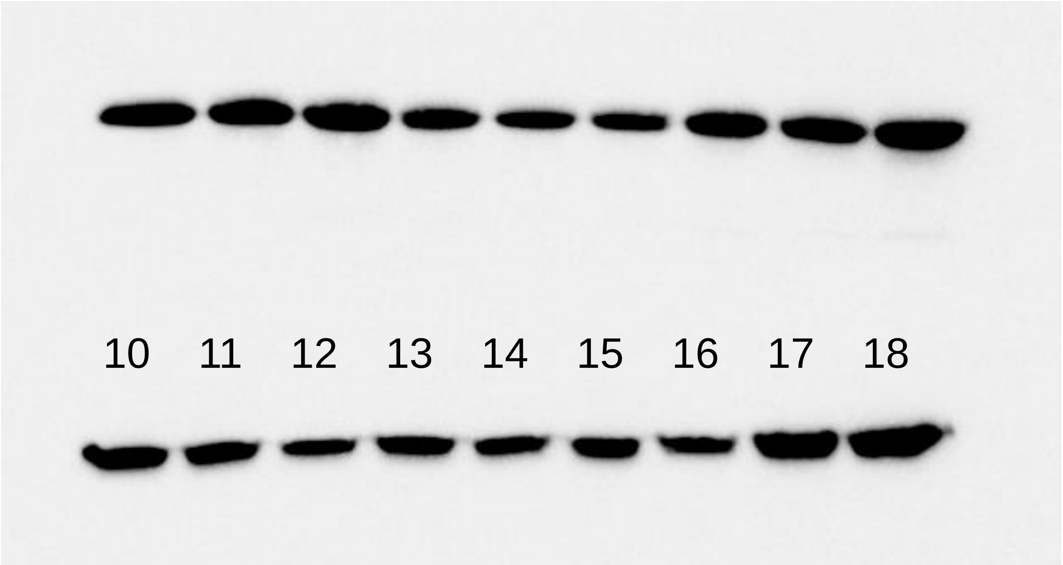

HPRT

HPRT

1: I/R group at day 1

2: I/R group at day 3

3: I/R group at day 5

4: I/R group at day 7

5: Rat kidney (positive control)

6: I/R group at day 10

7: Non-I/R group at day 1

8: Non-I/R group at day 3

9: Rat kidney

10: Non-I/R group at day 5

11: Non-I/R group at day 7

12: Non-I/R group at day 10

13: Control group at day 1

14: Control group at day 3

15: Control group at day 5

16: Control group at day 7

17: Rat kidney

18: Rat kidney

Supplementary Figure S1.

The complete (unedited) Western blot images provided in the manuscript.

APN/CD 13

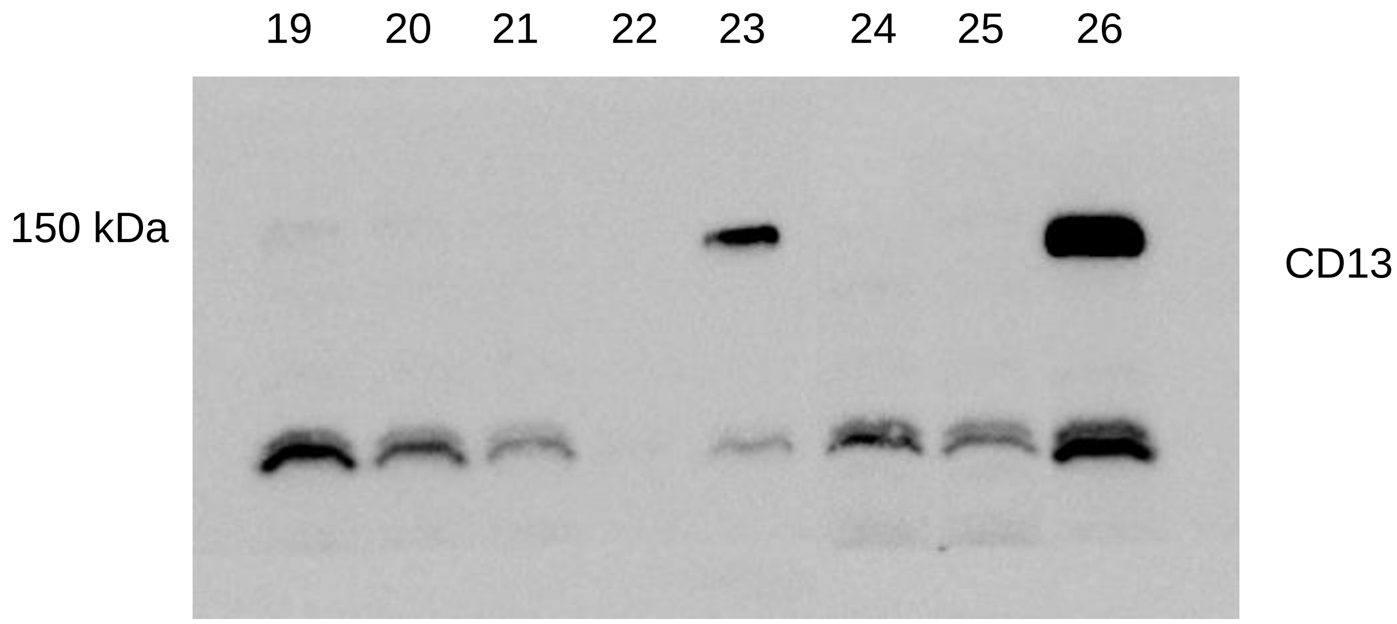

HPRT housekeeping gene

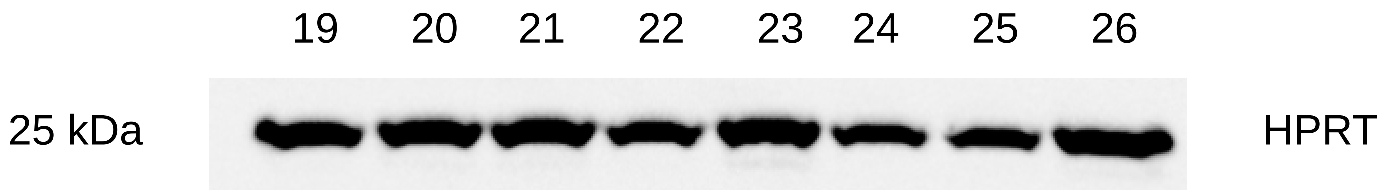

19: Control group at day 10

20: Control group at day 1

21: Control group at day 3

22: Control group at day 5

23: Rat kidney

24: Control group at day 7

25: Control group at day 10

26: Rat kidney
